# Clinical Outcome of Posterior Instrumented Stabilization and Transpedicular Decompression in Patients Presenting with Thoracic or Lumbar Spinal Tuberculosis in a Tertiary Care Center: A Descriptive Cross-sectional Study

**DOI:** 10.31729/jnma.8179

**Published:** 2024-08-31

**Authors:** Dinesh Bhandari, Sabin Pokharel, Praja Pokharel, Sushil Paudel, Dinesh Kafle, Rohit Kumar Pokharel

**Affiliations:** 1 Department of Orthopedics and Trauma Surgery, Maharaigunj Medical Campus, Tribhuvan University Teaching Hospital, Maharajgunj, Kathmandu, Nepal; 2 Department of Orthopedics, National Trauma Center, Mahaboudha, Kathmandu, Nepal; 3 Department of Public Health, Kathmandu University School of Medical Sciences, Dhulikhel, Kavre, Nepal

**Keywords:** *back pain*, *kyphosis*, *neurologic dysfunctions*, *Pott's disease*, *spinal tuberculosis*

## Abstract

**Introduction:**

Posterior instrumented stabilization is a commonly done surgery in spinal tuberculosis. This study aims to evaluate the clinical, radiological, and neurological outcomes of posterior instrumented stabilization and transpedicular decompression in thoracic and lumbar spinal tuberculosis.

**Methods:**

A descriptive cross-sectional study was conducted for one and a half years with at least six months of follow-up in a tertiary care center. The study was approved by the Institutional Review Committee (Reference number: 119 (6-11-5) 2/075-076). Total sampling was done and the study included patients over 18 years of age with spinal tuberculosis of the thoracic or lumbar regions. These patients underwent posterior instrumented stabilization and transpedicular decompression at the tertiary care center. The age, site of involvement, Visual Analog Scale score for back pain, neurological status as per Frankel Neurology grading, and local kyphotic angle in X-ray were recorded. The median, interquartile range and percentage were calculated. The data was entered in Microsoft Excel 2016 and analysis was done using Epi Info software version 7.2.

**Results:**

Thoracic level was most commonly involved in 14 (46.68%) cases. The median back pain as assessed by the VAS score improved from 8 to 2 at the 6-month follow-up. There was improvement in the neurological grading of all cases and there was no loss of correction in the local kyphotic angle till the final follow-up. The median age of cases was 48 years (interquartile range: 35).

**Conclusions:**

Posterior instrumented stabilization and transpedicular decompression in adult patients with thoracic or lumbar spinal Tuberculosis achieves improvements in clinical, radiological, and neurological outcomes.

## INTRODUCTION

In recent Nepalese data, the National Tuberculosis Programme (NTP) registered 37,861 cases of all forms of Tuberculosis (TB) of which 10556 (28%) were extra-pulmonary cases.^1^ Spine is the commonest site of osteo-articular TB accounting for around 50% and the most frequent sites are thoracic and lumbar spine.^2^

Though multidrug Antitubercular therapy (ATT) is the mainstay of treatment for spinal TB, surgery is done in selected cases. This concept was popularized by Tuli as "the middle path regime".^2^ Posterior instrumented stabilization (PIS) and transpedicular decompression are commonly done surgery for spinal TB which provides circumferential decompression of neural elements along with three-column fixation by the same approach.^2-4^ This corrects the kyphosis and ensures long-term spinal stability.^3,5^

This study aims to evaluate the clinical, radiological, and neurological outcomes of PIS and transpedicular decompression in thoracic and lumbar spinal TB cases.

## METHODS

A descriptive cross-sectional study was conducted at the Department of Orthopaedics and Trauma Surgery, Tribhuvan University Teaching Hospital (TUTH) for a duration of one and a half years from September 2018 to March 2020 after approval from the Institutional Review Committee (119 (6-11-5) 2/075-076). Patients aged 18 years or more presenting to our center during the study period with the diagnosis of spinal TB involving thoracic or lumbar vertebra were included in the study. The diagnosis was based on clinical-radiological findings which included MRI findings as well.^6,7^ All these cases after routine examinations and investigations were started on ATT. Surgery cases were selected using the "middle path regime" of Tuli and surgery was considered in the presence of persistent marked pain despite ATT for two months, at initial presentation significant vertebral body destruction or kyphosis and progressive neurology deterioration or new onset deficit despite ATT.^8^ But the cases of kyphosis, neurological deficit (paraplegia of late-onset) due to healed TB lesions, bony internal gibbus, multi-segmental disease with more than two vertebral destruction, marked intrinsic cord lesions, severely osteoporotic patients and cases not fit for surgery were excluded from the study as our procedure alone may not be sufficient to address these.^5^ Total sampling technique was employed and all the cases satisfying the inclusion criteria and undergoing surgery during the study period were included in the study.

After taking the informed written consent, cases of spinal TB with indications of surgery were scheduled for operation. Before the surgery, all cases were started on ATT at least for two weeks. The age, site of involvement, Visual Analog Scale (VAS) score for back pain, neurological status as per Frankel Neurology grading, and Local kyphotic angle (LKA) in X-ray (measured as per the Dickson method where lines are drawn posterior to vertebra above and below on lateral view) are recorded on the proforma by the principal investigator.^9,10^ Infected materials taken during the surgery were sent for histopathological examination and geneXpert testing.

The patient was mobilized as per pain tolerance with spinal braces under physiotherapist guidance from postoperative day one for ambulant patients. For non-ambulant patients, wheelchair mobilization was done. Postoperative physiotherapy was done to ensure early mobilization. ATT was continued for a total of 18 months duration with an initiation phase of 3 months and a continuation phase of 15 months.^11^ The cases were followed up and VAS scores for back pain, neurological status, and LKA were recorded on the proforma on postoperative day 1, 3 months, and 6 months respectively by the principal investigator.

The data was entered in Microsoft Excel 2016 and analysis was done using Epi Info software version 7.2. The median, interquartile range (IQR) and percentage were calculated.

## RESULTS

A total of 49 patients were diagnosed with spinal TB during the study period. Among them, 18 (36.73%) cases were managed conservatively and 31 (63.27%) cases underwent surgery. Out of patients who underwent surgery, 1 (2.04%) patient lost to follow-up, therefore a total of 30 (61.22%) patients were included in the study. The cases enrolled were between 18 and 73 years old (median 48 years, IQR 35). There were 14 (46.68%) cases, with thoracic-level involvement (Table 1).

**Table 1:** General characteristics of patients' who underwent surgery (n= 30).

General Characteristics	n (%)
**Gender**	
Male	17 (56.67)
Female	13 (43.33)
**Spinal Level involved**	
Thoracic	14 (46.68)
Thoracolumbar junction (T12-L1)	8 (26.66)
Lumbar	8 (26.66)

There was no recurrence until the final follow-ups in all 30 (100%) cases. All 30 (100%) of the cases had histopathological reports suggestive of TB. Multi-drug resistant (MDR) TB was observed in one case (3.33%) diagnosed by geneXpert testing and was started on ATT for multi-drug resistant TB as per the national guideline.

The pain as assessed by the VAS score improved from 8 to 2 at the 6-month follow-up. No loss of correction in LKA was seen at 6 months of follow-up (Table 2).

**Table 2:** Comparisons of Visual Analog Scale score and Local Kyphotic Angle measured on X-ray at different times of assessment (n = 30)

Variables	Median	IQR
**Visual Analog Scale score**		
Preoperative	8	1
Postoperative day 1	6	2
3 months follow up	4	1
6 months follow up	2	1
**Local Kyphotic Angles (Degrees)**		
Preoperative	18	12
Postoperative day 1	11	8
3 months follow up	8	6
6 months follow up	7.50	5

At three months of follow up 3 (10%) patients with Frankel A improved to Frankel B. Nine (30%) patients with Frankel E didn't show further neurological deterioration (Table 3). At the final follow-up of 6 months, cases improved to Frankel D and Frankel E. Superficial wound infection was observed in 2 (6.67%) which were were managed conservatively by regular dressing and the addition of antibiotics. Misalignment of 1 (3.33%) pedicle screw while instrumenting the thoracic vertebra was observed.

**Table 3:** Comparisons of neurology as Frankel neu-rology grading at different times of assessment (n = 30).

Frankel neurology grade	Preoperative n (%)	Postoperative		
		Day 1 n (%)	3 months n (%)	6 months n (%)
A	3 (10)	3 (10)	-	-
B	5 (16.67)	4 (13.33)	3 (10)	-
C	8 (26.67)	9 (30)	3 (10)	-
D	5 (16.67)	5 (16.67)	6 (20)	7 (23.33)
E	9 (30)	9 (30)	18 (60)	23 (76.67)
Total	30 (100)	30 (100)	30 (100)	30 (100)

## DISCUSSION

Tuberculosis of the spine is a common condition that we frequently encounter in our setting.^2,12^ Among the different surgical options available in selected cases posterior instrumented stabilization and transpedicular decompression are routinely done for these cases when indicated.^3-5^-^13^-^14^ These procedures done under the cover of ATT showed significant improvement in VAS score, neurology, and correction of LKA in our study.

In our study, the median age of presentation was 48 years and more than half of the total cases were male. This was similar to the study by Patidar et al. done in the Indian population where 16 (80%) cases were male and 4 (20%) were female out of 20 patients with a mean age of 37.9 years (range 16-57 years).^7^ Most of the previous studies have the mean age at the 3^rd^ to 4^th^ decade of life.^4,6,13^ Tuberculosis can occur at any age but tends to become more common at extreme ages. In our study, 9 out of 30 patients were more than 60 years old. A study done on the Taiwanese population found that more than 50% of the cases were over the age of 65 and attributed this finding to a longer life expectancy in comparison to other countries.^15^ The high occurrence of tuberculosis in old age is attributable to compromised immune responses resulting from increased co-morbidities. Older adults are more likely to develop atypical forms of tuberculosis such as tuberculosis meningitis, renal or skeletal disease, or sputum smear-negative pulmonary involvement.^16^

The thoracic spine is the most common site for the involvement of skeletal tuberculosis overall followed by the lumbar and thoracolumbar spines.^2,12,17^ Our study had similar findings. However some studies have shown more frequent involvement of the lumbar spine in spinal TB than the thoracic spine which is different from our findings.^15,18^

The median VAS score in our study shows gradual improvement with a decreasing trend with the time of follow-ups. A K Jain et al. in a similar study found preoperative VAS scores of 8.7, postoperatively VAS of 3.1, 3-month VAS of 2.7, and 6-month 2.1 respectively.^4^ Our study findings were similar to the findings of other studies showing a gradual improvement.^7^ The study by Liu et al. reports the postoperative VAS score at 6 months follow-up to be less than our study.^6^ The cases in this study had a lower preoperative VAS score than our study; hence, the postoperative score must have been on the lower side. Also, this is a subjective score and due to differences in lifestyle perceptions of pain may differ among individuals.

Localized kyphosis is present in up to 80% of cases of spinal TB patients; only 3-5% progress to greater than 60 degrees.^12^ There was no loss of correction in LKA at the final 6-month follow-up in our study, which was different from the findings of Liu et al. who reported loss amounting to 7.2+11.56 degrees but it was not statistically significant. This may be explained by the fact that Cobbs angle was used for calculating the kyphotic angle and bony fusion was achieved with bone graft in the study unlike our study and the follow-up period was also longer than our study.^6^ Another study with longer follow-up also shows a loss of correction of 4.1 degrees.^4^ A Study by Bezer et al. in cases of lumbosacral spondylitis undergoing surgery also shows statistically significant improvement in kyphotic angle from preoperative 17.5 to 5.4 degrees postoperatively during the 87 month follow but this study does not include cases of thoracic spondylitis as our study.^14^ So, for confirmation of the loss of corrections in our cases, a longer follow-up study may be required.

The cases in our study showed improvement in the neurology level and no deterioration of neurology was seen postoperatively. These findings were consistent with other studies done in different populations as well.^4-7,13,15^ Concomitant chemotherapy with ATT, along with adequate decompressive surgery may have resulted in an improved neurological outcome.

The study was done with a small sample size and shorter follow-ups than other studies. The scoring and measurement of LKA were done by the principal investigator which holds the possibility of observer bias. Since the outcome of our study is favorable in providing relief of back pain, correction of local kyphosis, and improvement in neurology PIS and transpedicular decompression in thoracic and lumbar spine tuberculosis in patients with surgical indications may be performed safely. However, a longer follow-up study with a larger sample size would further confirm the findings.

## CONCLUSIONS

Posterior instrumented stabilization and transpedicular decompression in adult patients with thoracic or lumbar spinal tuberculosis achieve improvements in clinical, radiological, and neurological outcomes.

## References

[1] GoN, DoHS. (2021/22). Annual Report 2078/79 (2021/22) TB Part [Internet]..

[2] Rajasekaran S, Soundararajan DC, Shetty AP, Kanna RM (2018). Spinal tuberculosis: current concepts.. Global Spine Journal..

[3] Varatharajah S, Charles YP, Buy X, Walter A, Steib JP (2014). Update on the surgical management of Pott's disease.. Orthopaedics & Traumatology: Surgery & Research..

[4] Jain A, Jain R, Kiyawat V (2016). Evaluation of outcome of posterior decompression and instrumented fusion in lumbar and lumbosacral tuberculosis.. Clinics in Orthopedic Surgery..

[5] Kumar MN, Joseph B, Manur R (2013). Isolated posterior instrumentation for selected cases of thoracolumbar spinal tuberculosis without anterior instrumentation and without anterior or posterior bone grafting.. European Spine Journal..

[6] Liu Z, Liu J, Peng A, Long X, Yang D, Huang S (2014). One-stage posterior debridement and transpedicular screw fixation for treating monosegmental thoracic and lumbar spinal tuberculosis in adults.. The Scientific World Journal..

[7] Patidar AB, Mehta RP, Sharma SK, Vyas GB, Singh V, Ramchandra O (2017). Single-stage posterior-only debridement and transpedicular screw fixation for dorsolumbar tuberculosis: A prospective study of twenty cases.. Journal of Orthopaedics and Spine..

[8] Tuli SM (2016). Tuberculosis of the skeletal system [Internet]..

[9] Dickson JA (1967). Spinal tuberculosis in Nigerian children: a review of ambulant treatment.. The Journal of Bone & Joint Surgery British Volume..

[10] Maynard FM, Bracken MB, Creasey G, Donovan WH, Ducker TB, Garber SL (1997). International standards for neurological and functional classification of spinal cord injury.. Spinal cord..

[11] Pokharel RK (2006). Anti-tubercular treatment regime for Musculoskeletal Tuberculosis.. JNMA J Nepal Med Assoc..

[12] Spiegel DA, Singh GK, Banskota AK (2005). Tuberculosis of the musculoskeletal system.. Techniques in Orthopaedics..

[13] Biakto KT, Arifin J, Lau H, Lee J (2014). Evaluation Outcome Of Posterior Decompression With Instrumented Fusion In Thoracic And Thoracolumbar Tuberculosis.. infection.

[14] Bezer M, Kucukdurmaz F, Aydin N, Kocaoglu B, Guven O (2005). Tuberculous spondylitis of the lumbosacral region: long-term follow-up of patients treated by chemotherapy, transpedicular drainage, posterior instrumentation, and fusion.. Clinical Spine Surgery..

[15] Weng CY, Chi CY, Shih PJ, Ho CM, Lin PC, Chou CH (2010). Spinal tuberculosis in non-HIV-infected patients: 10-year experience of a medical center in central Taiwan.. Journal of Microbiology, Immunology and Infection..

[16] Golden MP, Vikram HR (2005). Extrapulmonary tuberculosis: an overview.. American family physician..

[17] Tuli SM (2007). Tuberculosis of the spine: a historical review.. Clinical Orthopaedics and Related Research®..

[18] Pertuiset E, Beaudreuil J, Lioté F, Horusitzky A, Kemiche F, Richette P (1999). Spinal tuberculosis in adults: a study of 103 cases in a developed country, 1980-1994.. Medicine..

